# Late effects after allogeneic hematopoietic stem cell transplantation in patients with primary immunodeficiency

**DOI:** 10.3389/fimmu.2026.1752700

**Published:** 2026-07-01

**Authors:** Gintė Grubliauskaitė, Audronė Mulevičienė, Jelena Rascon

**Affiliations:** 1 Faculty of Medicine, Vilnius University, Vilnius, Lithuania; 2 Center for Pediatric Oncology and Hematology, Vilnius University Hospital Santaros Klinikos, Vilnius, Lithuania

**Keywords:** hematopoietic stem cell transplantation, inborn errors of immunity, late effects, long-term outcomes, primary immunodeficiency

## Abstract

**Background:**

Allogeneic hematopoietic stem cell transplantation (HSCT) has markedly improved survival in children with primary immunodeficiencies (PIDs) transforming previously fatal diseases into curable conditions. As survival improves late effects are emerging as major concerns. We performed a systematic review aiming to identify, categorize, and summarize late effects (LEs) following allogeneic HSCT in pediatric patients with PIDs.

**Methods:**

Following PRISMA guidelines, PubMed and EBSCOhost databases were searched without date restrictions for studies reporting LEs in patients who underwent allogeneic HSCT for PIDs at the age of 0–17 years and survived at least 2 years after HSCT.

**Results:**

Of 1,142 screened publications, 31 met the inclusion criteria, including 2,293 patients transplanted from 1981 to 2019 and followed for long-term outcomes. The main indications included severe combined immunodeficiency, Wiskott-Aldrich Syndrome (WAS), chronic granulomatous disease, leukocyte adhesion deficiency, and other rare PIDs. At least one LE was reported in 544 out of 2,293 patients (24%) who were followed up from 2 to 38 years. Growth and developmental delay were the most frequently reported LEs, observed in at least 280 patients (12.2%). This was followed by neurological (n=173, 7.5%) and autoimmune or hematologic (n=161, 7.0%) long-term issues. Pulmonary and skin complications were noted in 150 patients each (6.5% each), while dental and skeletal impairments affected 112 patients (4.9%). Endocrine issues were reported in 99 patients (4.3%), and gastrointestinal or liver impairments occurred in 75 patients (3.3%). Secondary malignancies, renal/metabolic, recurrent infections, hearing loss, ocular and vascular late complications were rare and reported in less than 1% of patients.

**Conclusions:**

A substantial proportion of pediatric HSCT survivors with PIDs experience LEs. They affect various organs and systems years after an immune system replacement. These findings highlight the need for lifelong, multidisciplinary follow-up to optimize long-term health, functional outcomes, and quality of life.

**Systematic review registration:**

https://www.crd.york.ac.uk/PROSPERO/view/CRD42024621972, identifier CRD42024621972.

## Introduction

Allogeneic hematopoietic stem cell transplantation (HSCT) has significantly improved the prognosis of children with primary immunodeficiencies (PIDs). Conditions once uniformly fatal – such as severe combined immunodeficiency (SCID) and related T- or B-cell disorders – are now often cured by immune system replacement. Over the last two decades, the number of allogeneic HSCTs has been constantly increasing, including the use of haploidentical stem cell sources (1). Therefore, long-term outcomes become of special interest. Moreover, the number of inborn errors of immunity (IEI) being identified is growing, largely due to advances in molecular diagnostics and increased clinical awareness (2). Allogeneic HSCT offers a potentially curative approach for newly diagnosed IEI; however, the late effect (LE) burden is important when deciding to proceed to transplant.

Outcomes after HSCT for PIDs have improved substantially in recent years, with modern cohorts reporting approximately 80% 5-year overall survival (3). Nonetheless, survival is an insufficient outcome measure of the ultimate success of HSCT. Many survivors develop LEs related either to the underlying disease and pre-transplant complications or due to the transplant-related toxicity. Frequently reported issues include endocrine problems – thyroid dysfunction, growth failure, and delayed puberty (4–6), neurocognitive difficulties (7, 8), autoimmunity and inflammation (3), skeletal abnormalities (9, 10), and, rarely, secondary malignancies (11). Risks for LE development vary by genetic defect causing the underlying PID, conditioning regimen, and age at HSCT (8, 12), they accumulate with time so that LEs can emerge years after transplant (7). Importantly, LEs may be influenced by pre-transplant manifestation of the underlying PID and its management. Infections and immune dysregulation before transplantation can cause irreversible organ damage. Therefore, delayed primary diagnosis and transplantation may further increase the risk of long-term effects (13).

Many centers conducted retrospective reviews or followed late outcomes prospectively in stem cell recipients who received allogeneic HSCT to correct an IEI. The number of publications addressing post-transplant LEs is constantly increasing. We conducted a systematic review of available evidence to map LEs after allogeneic HSCT in children with PID. We aimed to identify the reported long-term complications and their frequency, so that clinicians and families can anticipate, monitor, and manage them more effectively.

## Methods

The systematic review was conducted in accordance with the Preferred Reporting Items for Systematic Reviews and Meta-Analyses (PRISMA) guidelines (14). The study protocol was registered in the International Prospective Register of Systematic Reviews (PROSPERO) (registration number: CRD42024621972) before the search.

### Eligibility criteria

The inclusion criteria were pediatric patients with PID who underwent allogeneic HSCT at ages 0–17 years. Studies reporting HSCT for PID conducted beyond age 18 were excluded. Cohorts that were mixed (pediatric and adult) or lacked PID-specific post-HSCT data were also excluded from the total. We included only full-text scientific publications of all types. Recurring scientific publications, partial-text scientific articles, and articles not written in English were excluded. LEs were defined as complications that occurred or persisted ≥2 years after HSCT as defined by Wauben et al. (15). A minimal 2-year reported follow-up was an inclusion criterion for the selected publications.

### Literature search

The *PubMed* and *EBSCOhost* database were searched using the following keywords and their combinations: ((“SCID”[All Fields] OR “Severe combined immunodeficiency”[All Fields] OR “Severe combined immunodeficiencies”[All Fields] OR “Severe combined immunodeficiency disease”[All Fields] OR “Severe combined immune deficiency”[All Fields] OR “Severe combined immune deficiencies”[All Fields] OR “Inborn errors of immunity”[All Fields] OR “Inborn errors immunity”[All Fields] OR “Primary immunodeficiency”[All Fields] OR “Primary immunodeficiencies”[All Fields] OR “Primary immunodeficiency disorder”[All Fields] OR “Primary immunodeficiency disorders”[All Fields] OR “Primary immunodeficiency disease”[All Fields] OR “Primary immunodeficiency diseases”[All Fields]) AND (“Bone marrow transplant”[All Fields] OR “Bone marrow transplantation”[All Fields] OR “Allogeneic bone marrow transplantation”[All Fields] OR “Pediatric bone marrow transplant”[All Fields] OR “Hematopoietic stem cell transplant”[All Fields] OR “Hematopoietic stem cell transplantation”[All Fields] OR “Allogeneic hematopoietic stem cell transplantation”[All Fields] OR “Allogeneic stem cell transplant”[All Fields] OR “Allogeneic stem cell transplantation”[All Fields] OR “Cord blood stem cell transplantation”[All Fields])) AND (“Late effects”[All Fields] OR “result*”[All Fields] OR “outcome*”[All Fields] OR “complication*”[All Fields]). The literature selection was not limited by publication date. The search was performed on 15 April 2024. Automatic search tools were used to restrict results to studies involving humans, pediatric populations (birth to 18 years), and articles published in English. G. G. conducted a literature search.

### Study selection and data extraction

Deduplication of found articles was performed with the Systematic Review Management Platform Rayyan (http://rayyan.qcri.org). First, the titles and abstracts were screened for eligibility, followed by a full-text review of the potentially included publications. Two reviewers (G. G. and A. M.) independently screened and selected the eligible articles. Team members compared the chosen publications and extracted data, resolving any disagreements engaging the third reviewer (J. R.) and through discussion among the entire team.

**Figure 1:**
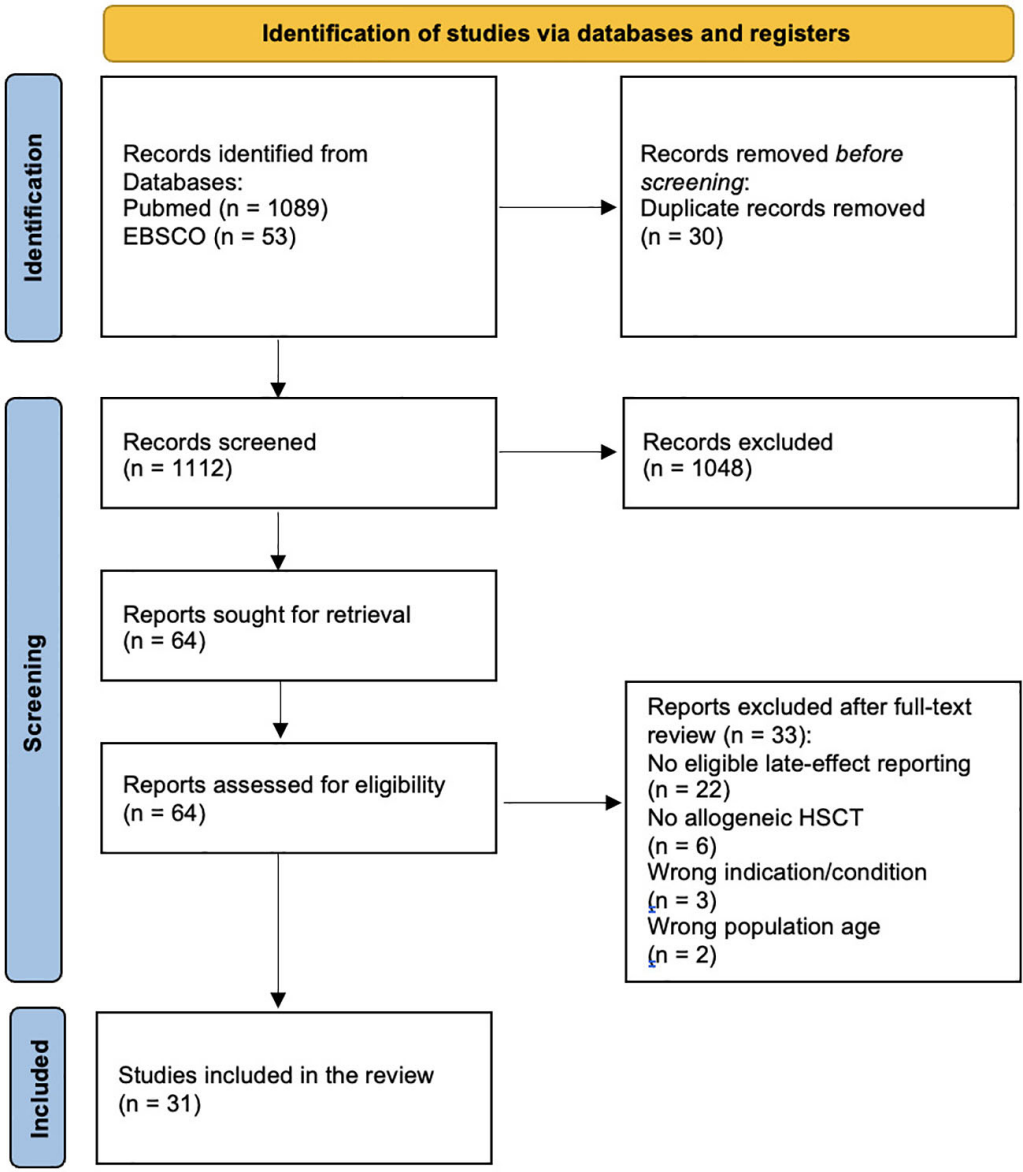
PRISMA 2020 flow diagram of study selection.

Of the 1,142 publications acquired from the search, 64 were included after abstract evaluation for full-text review. Thirty-three articles were excluded because they did not meet the inclusion criteria or did not contain relevant information. In total, 31 studies were eligible for the review (**Figure 1**). Two reviewers (G. G. and A. M.) independently extracted study characteristics and outcome data; discrepancies were resolved through discussion and consensus. LEs were grouped based on the most frequently reported affected organ or system. For quantitative reporting, outcomes were summarized as absolute numbers and percentages. All data were extracted from the main manuscripts, primary tables/figures, and supplemental datasets where provided. The included studies were highly heterogeneous with respect to design, patient populations, transplant indications, conditioning regimens, follow-up duration, and definitions/reporting of late effects. Due to this heterogeneity and inconsistent reporting, results were synthesized descriptively.

## Results

### Study characteristics and synthesis

Thirty-one studies that met the inclusion criteria for this systematic review reported 3355 patients who received an allogeneic graft to correct a PID. Overall, 2293 patients out of 3355 recipients survived, were followed up for at least 2 years, and screened for LE. The details on the selected 31 studies are summarized in **Supplementary Table 1**. The most common PIDs reversed by allogeneic transplantation were SCID, Wiskott-Aldrich syndrome (WAS), chronic granulomatous disease, leukocyte adhesion defect, and other rare PIDs. The transplantation period spanned from 1981 to 2019 (16, 17). Follow-up across all studies ranged from 2 to 38 years and was explicitly stated in 29 out of 31 articles. Both single-center and multicenter cohorts met the inclusion criteria, as well as three case reports and three case series (5, 10, 18–21). The total number of patients followed for more than 2 years ranged from 1 (21) to 596 (3) per publication. The median age at first HSCT ranged from 1.5 months (19) to 36 months (6) and was reported in 21 of 31 studies (3–8, 10–12, 16, 18, 22–28).

Genotype reporting differed considerably across the included studies, ranging from detailed gene-specific series to mixed cohorts in which genotype was not specified. The most frequent gene defects were IL2RG, ADA, and JAK3, followed by DNA-repair–associated disorders (**Table 1**). Conditioning approaches were also heterogeneous. Conditioning strategies varied substantially across studies. Unconditioned transplantation and busulfan-based conditioning were both frequently reported, while treosulfan-based regimens were reported less often. Many cohorts included more than one conditioning approach; exclusively unconditioned or exclusively conditioned cohorts were less common. In addition, some cohorts included patients who received more than one allogeneic graft, and several studies reported the use of adjunct therapies such as enzyme replacement therapy (**Table 1**; **Supplementary Table 1**).

**Table 1:** Genotype and conditioning reported across included publications (n=31).

Category	Number of studies reported respective data	References
Genotype		
IL2RG	13	(4, 7, 8, 11, 13, 16, 22, 23, 28–30, 32, 33)
ADA	13	(4, 7, 8, 11, 13, 17, 18, 22, 29, 30, 32–34)
JAK3	11	(4, 7, 8, 13, 17, 23, 24, 29, 30, 32, 33)
DNA–repair–associated defects	11	(4, 8, 11, 13, 19, 24, 27, 29, 30, 32, 33)
WAS	8	(3, 4, 6, 9, 21, 30, 31, 35)
CGD	4	(3, 4, 6, 9)
Genotype not stated	6	(3, 5, 9, 25, 31, 35)
Conditioning		
Unconditioned HSCT reported	19	(3, 4, 6–8, 11–13, 16–20, 22–24, 30, 32, 33)
Busulfan-based	17	(4, 6, 8, 9, 11, 18, 22–28, 30–32, 34)
Treosulfan-based	6	(6, 9, 10, 21, 23, 24)
TBI exposure	4	(11, 22, 31, 35)
Mixed conditioning (≥2 approaches per cohort)	17	(3–5, 7, 8, 11, 18, 21, 23, 24, 26, 28–32, 34)
Unconditioned-only cohorts	5	(13, 16, 17, 19, 20)
Conditioned-only cohorts	10	(5, 9, 10, 21, 25–27, 29, 31, 35)
GvHD		
Acute GvHD	17	(3–6, 8, 9, 11, 12, 16, 18, 19, 22, 26–31)
Chronic GvHD	15	(3, 4, 6, 7, 9, 11–13, 16, 18, 23, 27–31)

Graft-versus-Host disease (GVHD) reporting was incomplete and heterogeneous in explicitness. Acute and chronic GVHD reporting overlapped: acute GVHD was reported in 17 of 31 publications and chronic GVHD in 15 of 31 publications; these categories were not mutually exclusive because several publications reported both, as summarized in **Table 1**.

**Figure 2:**
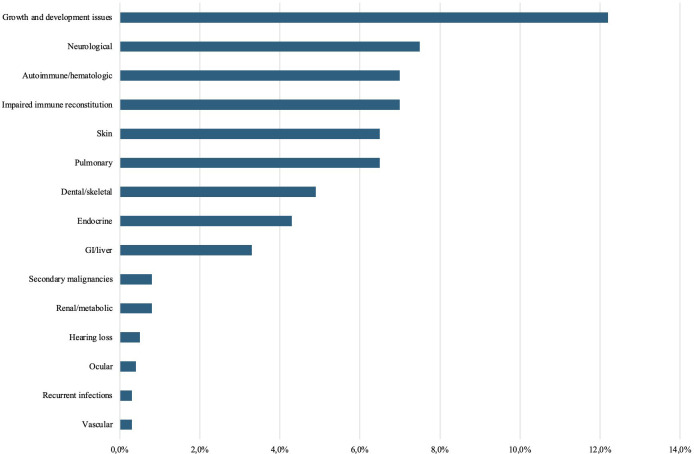
Reported late-effect domains after allogeneic HSCT in pediatric PID survivors.

Of the 2,293 included survivors, 544 (24%) had at least one LE. This number was retrieved directly from six publications whenever explicitly stated (**Table 2**). In the studies that did not report the total number of patients with at least one LE, the highest reported patient number within the most frequent single late-effect category was used as a conservative estimate of the minimum number of affected patients in the described cohort (**Table 2**). Various organs, systems, and functions appeared to be affected late after HSCT, as summarized in **Table 2**. Impairment of growth and development was the predominant long-term problem documented in 21 of 31 included studies. Growth and developmental LEs accounted for 280 reported patients, corresponding to 12.2% of the 2,293 included survivors (**Figure 2**). It was followed by neurological (n=173, 7.5%) and autoimmune/hematologic (n=161, 7.0%) LEs. Pulmonary and skin complications were also commonly reported (each n=150, 6.5%), while dental and skeletal (n=112, 4.9%), endocrine (n=99, 4.3%), and gastrointestinal (including liver) dysfunction were reported less frequently (n=75, 3.3%). Secondary malignancies, renal and metabolic complications, recurrent infections, hearing loss, ocular and vascular late complications were rare and reported in less than 1% of patients (**Table 2**; **Figure 2**).

**Table 2:** Reported late effects after allogeneic HSCT in patients with primary immunodeficiency. Cell values indicate the number of patients with each late effect domain.

Included studies (reference number)	Number of transplanted patients	Included into present systematic review	Number of patients to have at least one LE	Late effect															Percentage of patients reported to have at least one LE in the study
				Growth and development issues	Neurological	Autoimmune/hematologic	Impaired immune reconstitution	Skin	Pulmonary	Dental/skeletal	Endocrine	GI/liver	Secondary malignancies	Renal/metabolic	Recurrent infections	Hearing loss	Ocular	Vascular	
Abd Hamid et al., 2017 (23)	43	31	21*	3		2	15	9	3						1			2	68%
Abd Hamid et al., 2018 (24)	38	30	30	7		3	4	4	3	3	2			1		1			100%
Botto et al., 2021 (10)	7	6	6*	6			1			6			1	1		2	2		100%
Çağdaş et al., 2012 (19)	2	2	2	2			1												100%
Chou et al., 1996 (35)	15	15	15*	16					2	4	1		1	2			1		100%
Coppola et al., 2023 (21)	1	1	1										1						100%
Cuvelier et al., 2016 (12)	8	8	2	2			1	3			4			3				2	25%
de Kloet et al., 2022 (6)	315	74	11								18								15%
DiNardo et al., 2012 (30)	39	39	10		1	3	6	1		1	1								26%
Eissa et al., 2024 (7)	662	399	142*	31	34	6		8	26	54	15	23	9	1					36%
Golwala et al., 2023 (9)	429	340	32	6						32			1						9%
Grunebaum et al., 2006 (29)	105	56	3		3										1				5%
Hardin et al., 2022 (17)	177	88	44	59	30	0	62	36	32										50%
Hönig et al., 2007 (18)	15	12	7		30														58%
Lin et al., 2009 (25)	16	16	16	16	16														100%
Lum et al., 2021 (3)	596	596	31		5	6					19			1					5%
Mazzolari et al., 2005 (26)	11	9	2		3	2											1		22%
Mazzolari et al., 2007 (8)	58	40	7	12	6		5			3	7					2			18%
Mazzolari et al., 2009 (33)	74	49	9	11	9	9		9	3		5			1	4				18%
Miyamoto et al., 2021 (11)	181	181	24	24					1		7		2						13%
Nagasawa et al., 2017 (31)	74	29	3						3										10%
Nahum et al., 2009 (27)	6	6	4	4															67%
Neven et al., 2009 (32)	149	90	23*	22	1	11	4	24	8			18	2	3					26%
O’Marcaigh et al., 2001 (5)	16	11	3	3					2	2									27%
Patel et al., 2008 (16)	25	15	5	2	5	1		9	5	1	3	3				3	2		33%
Patel et al., 2009 (22)	23	13	4	1	2	2	2	2	7	1		1		2			1		31%
Railey et al., 2009 (13)	161	111	71*	48	19	3	59	41	51		5	24							64%
Roifman et al., 2008 (28)	10	10	3	3															30%
Schwaderer et al., 2005 (20)	2	2	2			1	2	2						2					100%
Scott et al., 2017 (34)	14	5	4	2	7	1		2	3	1	2	6	1	1	2	3	2	1	80%
Slatter et al., 2004 (4)	83	9	7								9								78%
Studies reporting, n (% out of 31)				21 (68)	15 (48)	17 (55)	11 (35)	13 (42)	15 (48)	11 (35)	15 (48)	6 (19)	8 (26)	11 (35)	4 (13)	5 (16)	6 (19)	3 (10)	
Total reported late-effects counts (sum across studies)	3355	2293	544	280	173	161	161	150	150	112	99	75	18	18	8	11	9	6	24%

LE, late effect.

*Studies evaluated the number of patients who had at least one LE. Blue shading indicates that the respective late-effect domain was reported in the study; unshaded cells indicate that no data for that domain were reported.

Based on the analyzed data, 1,749 survivors (76.3%) did not have any LE; however, this should be interpreted cautiously. As studies varied in cohort size, follow-up duration, and reporting practices, LE categories were not mutually exclusive (one patient may have had multiple LEs that was not possible to discriminate from available data). We benchmarked the LE pattern and percentage derived from 31 reviewed publications to the one reported in the five largest and most comprehensive studies that provided multi-domain data on LEs and included a substantial number of survivors with at least 2 years of follow-up (7, 11, 13, 17, 32). **Supplementary Figure 1A** illustrates a marked between-cohort variability in the frequency and distribution of reported LEs. Despite this heterogeneity, several consistent patterns emerged. Growth and developmental as well as autoimmune and hematologic LEs were the most frequent across the five largest cohorts, while pulmonary and skin complications were also recurrently reported. In contrast, renal and metabolic complications and secondary malignancies were uncommon across nearly all cohorts. The pooled data of 869 survivors also revealed that growth and developmental LEs remained the most frequent (21%). The pattern across five selected studies replicated the cumulative LE observed in the overall 31 studies (**Supplementary Figure 1B**). Overall, these findings show that although the absolute burden of specific LEs varied across cohorts, the dominant long-term morbidity pattern was driven by growth/developmental, immune-mediated, pulmonary, and skin complications.

### Late effects

#### Growth and developmental impairment

Impairment of growth and developmental impairment were particularly common among those who had received myeloablative conditioning or experienced chronic GVHD (6–9). Children who underwent HSCT for PID at a very young age – especially before age two – were more vulnerable to growth delays, particularly if they were already nutritionally compromised before transplant (8, 9). In some cases, late-onset growth hormone deficiency was suspected, although formal testing was not always performed or reported, leaving the full extent of endocrine involvement uncertain (9). For some patients, specific genetic diagnoses contributed directly to ongoing growth issues, e.g., children who had LIG4 deficiency continued to experience short stature, including two who had cartilage-hair hypoplasia.

#### Pulmonary complications

Pulmonary complications were described in 15 out of the 31 studies. Pathologic findings ranged from chronic sinusitis and bronchiectasis to more serious conditions, such as interstitial pneumonitis and late-onset non-infectious pulmonary complications (LONIPCs), often affecting individuals with suboptimal immune reconstitution, particularly those with JAK3 or IL2RG deficiencies (7, 8, 13, 32). Nagasawa et al. described a pediatric HSCT cohort in which 9 of 67 patients developed LONIPCs, including bronchiolitis obliterans and bronchiolitis obliterans organizing pneumonia in patients with Wiskott–Aldrich syndrome. MAC and chronic GVHD were associated with LONIPCs, whereas no LONIPCs occurred after reduced-intensity conditioning (31).

#### Autoimmune disorders

Autoimmune thyroiditis and hypothyroidism (both compensated and overt) were among the most common autoimmune complications. Some studies reported thyroid dysfunction in up to 62% of analyzed survivors, with higher manifestation in those experiencing chronic graft-versus-host disease (cGvHD) or lingering immune dysregulation (3, 4, 8, 12). The presence of anti-thyroid antibodies, such as anti-TPO, was frequently noted in these individuals (3, 4). Autoimmune cytopenias – especially autoimmune hemolytic anemia and immune thrombocytopenia – were another prominent LE. These were often observed in the context of chronic GvHD or incomplete immune reconstitution (3, 8, 12, 36). Though less common, a wide variety of other autoimmune disorders have also been reported, including rheumatological manifestations, psoriasis, myositis, alopecia areata, and vitiligo, many of which may have both medical and psychosocial consequences for young survivors (3, 8, 12).

#### Neurologic and cognitive complications

Neurological and cognitive complications were frequently documented among long-term survivors of HSCT for PID, particularly in those with early-onset or metabolically deficient disorders. Children with ADA-deficient SCID and other early-onset PIDs were especially vulnerable to motor and cognitive developmental delays (8, 18, 37). Neurocognitive and psychosocial difficulties – including learning/attention problems and emotional or behavioral symptoms – were reported in several cohorts of long-term survivors (7, 18, 25). In some cases, they might persist after early toxicity, e. g., one patient developed a long-lasting neurologic impairment following early post-HSCT encephalitis (33). Specific neurological signs – such as ataxia, hypotonia, and gait disturbances – were frequently observed in patients with immunometabolic syndromes (8, 18, 37). Hönig et al. found that as many as 40% of survivors experienced long-lasting neurologic or neurocognitive symptoms, including learning disabilities, behavioral challenges, hyperactivity, and sensorineural hearing loss. For most patients, there was no evidence of progressive neurological decline during later childhood or adolescence (18). This indicates that while early deficits are common, many children stabilize over time, especially with supportive care and early intervention services.

#### Secondary malignancies

While relatively rare, secondary malignancies were among the most worrisome long-term complications. Multicenter cohorts reported low but non-zero cumulative incidence of second malignancies in long-term survivors (7, 9). Across studies, the overall risk of cancer was higher in transplant survivors compared to age-matched controls, with reported cases including lymphomas, thyroid cancers, and non-melanoma skin cancers (7, 8, 21). In a large IEI survivors’ cohort bone tumors – both benign and malignant – were diagnosed: 9.4% developed non-osteopenic bone pathology; osteochondromas were most common, and one osteosarcoma occurred 13 years post-HSCT (9). A patient with WAS developed a Kaposiform hemangioendothelioma at age 5 and a desmoid tumor at age 10 - reminding us that patients may face multiple and rare tumor types over time (21). On average, secondary malignancies tended to emerge between 8 and 15 years after transplantation, well into long-term follow-up. Importantly, these malignancies were not limited to patients who had received total body irradiation. Several occurred in individuals treated with chemotherapy-only conditioning.

#### Recurrent infections

Recurrent bacterial infections, such as pneumonia and chronic sinusitis, were particularly common among patients with poor B-cell reconstitution, many of whom required long-term immunoglobulin replacement therapy to remain protected (7, 8, 23). Viral reactivations or persistent infections of CMV, EBV, and HPV caused interstitial pneumonitis and even secondary malignancies in some cases (7, 8, 23). Structural complications of the respiratory tract, such as sinusopathy and bronchiectasis, were observed in patients with delayed immune reconstitution or mixed donor chimerism, highlighting how prolonged immune dysfunction can manifest in long-term respiratory disease (8, 23, 24). Chronic GVHD, another immune-related complication, occurred in 15–20% of patients and often overlapped with autoimmune and infectious issues, compounding the overall burden of illness (7, 23).

#### Fertility impairment

The long-term effects on puberty and fertility varied across studies, but some patterns have emerged. Girls who received chemotherapy with alkylating agents, especially busulfan or cyclophosphamide, were more likely to experience hormonal issues in the long term. Approximately one in five female survivors went through delayed puberty or had amenorrhea, and there were cases of premature ovarian failure in girls treated with high-intensity regimens (3, 6, 7). Some male survivors were able to conceive children, suggesting that gonadal function can recover, at least partially, even after intensive treatment (7). Fertility was affected by the type of conditioning: those who received total body irradiation or high doses of busulfan faced a higher risk of infertility (3, 7). Overall, fertility data in this population were limited. The overall follow-up time for recipients transplanted in early infancy was insufficient.

## Discussion

For patients with PIDs, an allogeneic HSCT can be life-changing, often offering a healthier future. However, our systematic review, including 2,293 surviving recipients, showed that long-term complications may affect at least a quarter (24%) of survivors and extend well beyond two years after HSCT. Although the reviewed data showed that 1,749 survivors (76%) were LE-free, this should not be interpreted as a definitive absence of long-term sequelae. The cumulative number of patients affected by LEs (both overall and by LE category) may be even higher, as studies varied in cohort size, follow-up duration, and reporting practices; one patient might have several LEs that were not always carefully delineated. In addition, LEs may emerge far later than 2 years after HSCT. Thus, the actual LE burden might exceed 24%. The recently published literature review of LEs after HSCT in patients with hemophagocytic lymphohistiocytosis (HLH) reported the highly variable incidence, ranging from 7 to 52%. The cumulative incidence of clinically significant LE was 25% in those alive at 2 years, increasing to 41% at 15 years after HSCT (38).

The included studies cover 38 years – HSCT was performed from 1981 (16) to 2019 (17), during which HSCT practices and conditioning approaches evolved significantly. International IEI HSCT guidelines recommend different conditioning protocols across the myeloablative–reduced-intensity spectrum, depending on the underlying disorder and pre-transplant morbidity (39). Busulfan-based preparative regimens are considered myeloablative and should include therapeutic drug monitoring, whereas treosulfan may be a part of myeloablative or reduced-intensity approach, depending on the protocol used (39). Choice of conditioning strategy is crucial in PIDs when the underlying gene defect and pre-transplant morbidity may play a key role in the development of early toxicity and late complications. This is supported by Nagasawa et al., who reported LONIPCs after myeloablative conditioning but not after reduced-intensity conditioning (31). Only a minority of publications in our systematic review explicitly labeled conditioning intensity (MAC or RIC), limiting standardized intensity stratification across studies. This was an important limitation in our study. The available data suggest that tailoring conditioning intensity to the underlying PID and genetic diagnosis may help reduce long-term morbidity and warrant further investigation.

As summarized in **Supplementary Table 1**, the included articles were highly heterogeneous with respect to reported PID type, transplant approach, and duration of follow-up, which limited formal comparative analyses but allowed for the identification of consistent patterns of late morbidity. A key challenge across the reviewed evidence was disentangling the origin of LEs. The delineation whether the reported complications stemmed from genotype-driven biology of the underlying PID or persistence/progression of pre-transplant morbidity (e.g., infection-related organ damage, inflammatory complications), or reflected conditioning- and/or transplant-related toxicity per se was impossible. The reviewed cohorts frequently compiled multiple PID genotypes and transplant approaches. Most studies lacked standardized reporting of baseline organ function before HSCT, detailed conditioning exposure (including cumulative doses and therapeutic drug monitoring), and uniform definitions of LEs. An additional source of heterogeneity was cumulative exposure to toxicity-related risks across repeated transplants. In the Omenn syndrome cohort, most early transplants used myeloablative conditioning (MAC 10/11, RIC 1/11). Still, subsequent procedures used alternative approaches (e.g., immunosuppression-only or reduced-intensity after prior MAC, and MAC after prior RIC), meaning that patients who underwent multiple HSCTs were ultimately exposed to MAC at least once (26).

In our study, growth and developmental impairment were the most frequent LEs, accounting for 12.2% among all included survivors. Benchmarking against the five most comprehensive studies revealed that these LEs were also common, accounting for 21% (**Supplementary Figure 1B**). Although the study-specific analysis depicted on **Supplementary Figure 1A** shows that other organs and systems might be more frequently damaged. Ramme et al., who focused on LEs in patients transplanted for HLH, found that CNS-related LEs were the most frequent (31.0%), followed by growth hormone deficiency (16.5%) (38). CNS involvement is part of HLH manifestation; therefore, pre-transplant damage could contribute to late CNS complications and their highest incidence. We also found that persistent neurologic toxicity (ataxia, hypotonia, and gait disturbances) was more prominent in patients with immunometabolic IEI, where CNS impairment is usually present before HSCT (8, 18, 37). This underscores that LE patterns might be influenced by the patient inclusion and assessment criteria adopted by each study group. Therefore, a straightforward comparison of published evidence may be subject to selection bias.

Patients with DNA repair defects and immunometabolic disorders (Artemis, RAG1/2, LIG4, and adenosine deaminase deficiencies) more frequently exhibited growth impairment, endocrine dysfunction, and skeletal abnormalities during long-term follow-up (8–10, 18, 27). These complications were commonly reported in cohorts exposed to myeloablative conditioning regimens, particularly those including alkylating agents or total body irradiation, which are known to affect endocrine function, growth, and bone health (6, 7, 9, 35). However, in DNA-repair disorders, long-term growth and developmental burden may reflect the underlying disease biology rather than transplant toxicity alone. Studies including patients transplanted without conditioning or with reduced-intensity regimens more often described persistent immune dysregulation, mixed donor chimerism, and autoimmune complications, such as autoimmune cytopenias and thyroid disease. Across multiple cohorts, GVHD was an important modifier of long-term outcomes and was frequently associated with late autoimmune, endocrine, pulmonary, and dermatologic effects (3, 7, 8, 23). DiNardo et al. also illustrated that incomplete immune reconstitution may persist after unconditioned haploidentical transplantation, particularly in X-linked SCID, where long-term IVIG dependency was reported after unconditioned parental donor transplantation (30).

During the assembly and evaluation of selected studies, we encountered challenges arising from varying interpretations of LE categories, which directly affected the description of patients and LEs (7, 13, 17, 32). For instance, growth and developmental impairment might include growth hormone deficiency and neurocognitive development (4, 6, 7, 18, 25). In other studies, growth hormone deficiency could be attributed to endocrine disorders. This is an important limitation of our study that underscores the need for aligned LE categorizations and reporting guidelines (15, 39).

Health-related quality of life among survivors of allogeneic HSCT for PIDs demonstrates considerable variability and is influenced by ongoing treatment needs, late complications, and psychosocial adaptation. Studies report that survivors requiring continued therapies such as immunoglobulin replacement or prophylactic antibiotics experience significantly lower quality of life, particularly in emotional well-being, physical functioning, and school-related activities (16). The presence of chronic illness, notably autoimmune conditions and chronic GvHD, was consistently associated with poorer self-reported quality of life (7, 16). Therefore, there is an unmet need to reduce treatment-related toxicity, which contributes to LE burden. Recent advances in autologous hematopoietic stem cell gene therapy have provided an alternative curative option for selected primary immunodeficiencies, particularly X-linked SCID and related disorders. Published evidence indicates that effective immune reconstitution, non-involvement of donor immune cells, eliminates the GVHD-related complications, although long-term data on late effects remain limited (40).

## Conclusion

While allogeneic HSCT has greatly improved survival for patients with PIDs, this systematic review shows that a substantial proportion of survivors experience late complications. LEs range from growth, endocrine, and autoimmune disorders to infectious, neurocognitive, developmental, and malignant complications. They remain clinically significant and require lifelong surveillance.

The available data did not allow a systematic assessment of individual risk factors for LEs. However, the findings suggest that both the biological characteristics of the underlying PID, pre-transplant organ damage, and transplant-related factors contribute to the long-term morbidity profile observed after HSCT. This underlines the importance of individualized risk assessment and tailored long-term follow-up strategies that take genetic diagnosis, conditioning exposure, and post-transplant immune reconstitution into account. Multidisciplinary, coordinated follow-up is essential for the early detection and management of late effects, and continued research is needed to minimize their impact on future generations of HSCT recipients.

## Supplementary material

Supplementary Table 1 Characteristics of included articles.

Supplementary Figure 1 Late effects across the largest and most comprehensively reported pediatric PID HSCT cohorts.
