# Supplementary material for: Late effects after allogeneic hematopoietic stem cell transplantation in patients with primary immunodeficiency
Source: Front Immunol. 2026 Jul 1;17:1752700. doi: 10.3389/fimmu.2026.1752700 (PMC13368574; doi:10.3389/fimmu.2026.1752700)
Supplement: Supplementary Table 1 — Characteristics of included articles. [file Table1.docx]

**Supplemental Table S1.** Characteristics of included articles. The percentage was calculated from the number of patients included who met the inclusion criteria: survived more than 2 years after HSCT, eligible for the calculation of incidence of late effects (indicated in each respective cell)

| **Article, publication year (reference number)** | **Article type, country,**  **period of HSCT** | **Number of patients** | **PID types** | **Genetic defect** | **Age at first HSCT** | **Conditioning at first HSCT** | **Acute and chronic GvHD** | **Follow up** | **Reported late effects**  **(one individual may have >1 late effect)** | **Comments** |
| --- | --- | --- | --- | --- | --- | --- | --- | --- | --- | --- |
| Abd Hamid et al, 2017 (23) | Retrospective cohort study, United Kingdom,  1987–2012 | Transplanted  43  Alive  31  Included in this review  31 | SCID | N=31  IL2RG  25 (81%)  JAK3  6 (19%) | N=31  median 28 weeks  (range 2-52 weeks) | N=31  None  10 (32%)  Bu/Cy  12 (39%)  Campath/Flu/Treo  8 (26%)  Campath/Flu/Mel  1 (3%) | N=31  Chronic GVHD  not reported as a major late complication | N=31  >2 y post-HSCT | N=31  Ongoing immunoglobulin replacement  14 (45%)  Cutaneous warts  7 (23%)  Bronchiectasis  3 (10%)  Limb lymphoedema  2 (6%)  Delayed development  1 (3%)  Short stature  4 (13%)  Autoimmune hemolytic  anemia  1 (3%)  Recurrent otitis media  1 (3%)  Persistent neutropenia  1 (3%)  Autism spectrum disorder  1 (3%)  Dermatitis  1 (3%)  Psoriasis  1 (3%)  Specific  antibody deficiency to  pneumococcal antigen  1 (3%) | N=21 (68%) was reported to have persistent late effects |
| Abd Hamid et al., 2018 (24) | Retrospective longitudinal single-center cohort study, United Kingdom,  1988-2012 | Transplanted  38  Alive  30  Included in  This review  30 | SCID | N=30  IL7Rα  14 (47%)  Artemis  7 (23%)  RAG1  6 (20%)  RAG2  3 (10%) | N=30  Median 11,5 weeks (1–84 weeks) | N=30  None  5 (17%)  Bu/Cy  7 (23%)  Bu(oral)/Cy  4 (13%)  Bu/Cy/ATG  2 (7%)  Bu(oral)  1 (3%)  Campath/Treo/Flu  4 (13%)  Campath/  Treo/Cy  2 (7%)  Campath/Flu/Mel  2 (7%)  Treo/Cy  2 (7%)  Treo/Flu  1 (3%) | Not stated | N=30  >2 y post-HSCT | N=30  Short stature  7 (23%)  Ongoing immunoglobulin replacement  4 (13%)  Warts  4 (13%)  Dental issues  3 (10%)  Autoimmune hemolytic anemia  3 (10%)  Autoimmune hypothyroidism  2 (7%)  Bronchiectasis  2 (7%)  Hearing loss  1 (3%)  Chronic renal failure  1 (3%)  Chronic pulmonary disease  1 (3%) | A discrepancy was identified between Table 1 (n = 31) and the supplementary material (n = 30). After cross-checking the data, a numerical inconsistency was observed. In this table, we rely on the data presented in the supplementary material (n = 30) |
| Botto et al*.*, 2021 (10) | Multicenter retrospective case series, Italy, Germany, Sweden, Turkey  HSCT period not stated | Transplanted  7  Alive  7  Included in this review  6* | N=6  HLH  4 (67%)  SCID  1 (17%)  Omenn syndrome  1 (17%) | N=6  PRF1  2 (33%)  RAG1  2 (33%)  UNC13D  1 (17%)  UNK/NR  1 (17%) | N=6  Median 6.25 mo (5-20 mo) | N=6  Treo/TT/Flu/ATG  1 (17%)  Flu/Treo/Alemtuzumab  1 (17%)  Flu/Treo/ATG/VP  1 (17%)  Flu/Treo/VP  1 (17%)  Flu/Treo  1 (17%)  Flu/Treo/ATG  1 (17%) | N=6  GVHD  3 (50%) | Not explicitly stated | N=6  Chondrodysplasia  6 (100%)  Hearing abnormalities  2 (33%)  Papillary thyroid cancer  1 (17%)  Obesity  1 (17%)  Macrocephaly  1 (17%)  Hyperopia, astigmatism  1 (17%)  Mild myopia, astigmatism, bilateral small posterior lens opacities (cataracts)  1 (17%)  Functional asplenia  1 (17%) | *In one patient, HLH was secondary to leishmaniasis; due to its secondary etiology, this case was excluded from the systematic review. |
| Çağdaş et al., 2012 (19) | Case report,  Turkey,  HSCT period not stated | Transplanted  2  Alive  2  Included in this review  2 | N=2  SCID  2 (100%) | N=2  NHEJ1 deficiency  2 (100%)  Homozygous C622T mutation  2 (100%) | N=2  1.5 mo*  1 (50%)  15 mo  1 (50%) | N=2  None  2 (100%) | N=2  Acute intestinal GVHD  1 (50%) | N=2  7 y after HSCT  1 (50%)  1 y after HSCT  1 (50%) | N=2  Short stature  2 (100%)  Microcephaly  2 (100%)  Autoimmune hemolytic anemia  1 (50%) | *Reported as 1.5 months in the text; Table 1 appears to show 18 months. |
| Chou et al., 1996 (35) | Single center retrospective cohort study,  United States of America,  1982-1993 | Transplanted  15  Alive  8  Included in this review  15 (alive at 2 years after HSCT with reported delayed toxicities) | N=15  SCID  12 (80%)  WAS  2 (13%)  CHS  1 (7%) | Not stated | Not stated clearly | N=not stated clearly  Cy/ATG/TBI 7Gy  (8/15 patients had previously received unsuccessful HSCT without TBI) | N=15  GVHD  3 (20%) | N=15  Mean 6.6 y (0.3-11 y) | N=15  Diminished height  15 (100%)  Exostosis  4 (27%)  Chronic interstitial lung disease  2 (13%)  Mild chronic renal insufficiency  2 (13%)  Delayed puberty  1 (7%)  Bilateral cataract  1 (7%)  Secondary acute myelogenous leukemia  1 (7%) |  |
| Coppola et al., 2023 (21) | Case report,  Italy, year of HSCT not stated | Transplanted  1  Alive  1  Included in this review  1 | WAS | WAS gene mutation | 9 mo | Treo/Flu/ATG | No signs of GVHD | > 10 y | Kaposiform hemangioendothelioma at age 5 y  Desmoid tumor at age 10 y |  |
| Cuvelier et al., 2016 (12) | Retrospective, single-center cohort, Canada,  1992-2014 | Transplanted  8  Alive  8  Included in this review  8 | N=8  CID  8 (100%) | N=8  ZAP70 deficiency  8 (100%) | N=8  Median 7.25 mo (1 - 43 mo) | N=8  None  3 (37%)  Bu/Cy  3 (37%)  Rituximab and anti-thymocyte globulin  1 (13%)  Bu/flu and anti-thymocyte globulin  1 (13%) | N=8  Acute GVHD: grade II  3 (38%)  grade II  3 (38%)  Chronic GVHD: limited  (skin)  2 (25%) extensive2 (25%) | N=8  Median 13.5y (1.9-24y) | N=8  Chronic eczema  2 (25%)  Hypertension  2 (25%)  Premature  ovarian failure  2 (25%)  Decreased  height velocity  2 (25%)  Severe malnutrition and tube feeding  1 (13%)  Obesity  1 (13%)  Autoimmune alopecia areata  1 (13%)  Ongoing immunoglobulin replacement  1 (13%) |  |
| de Kloet et al., 2022 (6) | Single-center  retrospective cohort study, Netherlands  1997-2018 | Transplanted  315 (a mixed cohort of IEI and othe non-malignant disorders)  Alive  245  Described in a study  197  Included in this review  74* | N=74  SCID  20 (27%) HLH  15 (20%)  WAS  5 (7%)  CN  4 (5%)  CID  3 (4%)  CGD  3 (4%)  XLP  2 (3%)  CD40 deficiency  2 (3%)  DOCK8 deficiency  1 (1%)  AIE  1 (1%)  CD27 deficiency 1  (1%)  CHS  1 (1%)  FEL  1 (1%)  Hyper-IgE syndrome  1 (1%)  ICF syndrome  1 (1%)  IPEX syndrome  1 (1%)  LAD-1  1 (1%)  NOMID-like syndrome  1 (1%)  ZAP70 deficiency  1 (1%)  IEM  9 (12%) | Not stated | N=74  Median: 36 mo (11 - 80 mo) | N=74  Bu-based  50 (68%)  Treo-based  20 (27%)  Others  1 (1%)  None  3 (4%) | N=74  Acute GVHD Grade 0-I  62 (84%) Grade II-III 12 (16%) | Median 8.4 y (IQR 4.4 - 12.4 y) | Females with gonadal dysfunction  11/13 (85%)**  Males with gonadal  dysfunction  7/24 (29%)** | *De Kloet et al. included patients with inborn errors of metabolism in this group due to the small number of cases (9/74); there was no possibility to extract data only for IEI patients.  **Gonadal function was evaluated only in postpubertal females and males. |
| DiNardo et al., 2012 (30) | Retrospective single-center cohort study,  USA,  1986-2010 | Transplanted  39  Described in the study  39  Included in this review  39 | N=39  SCID  25 (64%)  Other PID  14 (36 %) | N=39  IL2RG  7 (18%)  IL7R  4 (10%)  ADA  3 (8%)  RAG1/2  2 (5%)  JAK3  1 (3%)  Other  16 (41%)  UNK/NR  6 (15%) | Not stated clearly | N=39  SCID (25):  None  8 (32%)  MAC  9 (36%)  RIC  8 (32%)  Other PID (14):  RIC  6 (43%)  MAC  9 (64%)* | N=39  SCID:  Acute GVHD  10 (40%)  Chronic GVHD  2 (8%)  Other PID:  Acute GHVD  7 (50%)  Chronic GVHD  4 (29%) | SCID:  Median 34.5 mo (3 – 145 mo)  Other PID:  Median 24 mo (1 – 326 mo) | N=39  Long-term IVIG replacement >2 y  6/25 (24%)  Autoimmune cytopenias  3/14 (21%)  Residual neurological deficit after CVA  1/25 (4%)  Infertility  1/14 (7%)  Chronic foot warts / dermatophytosis  1/14 (7%)  Spinal fracture  1/14 (7%) | *Reported in the article; totals exceed 100%, likely reflecting conditioning exposures/procedures rather than mutually exclusive patients. |
| Eissa et al., 2024 (7) | Retrospective multicenter cohort study, United States, Canada  1982-2012 | Transplanted 662  Alive >2 years  399  Included in this review  399 | N=399  SCID  399 (100%) | N=399  IL2RG  136 (34%)  JAK3  17 (4%)  ADA  20 (5%)  RAG1/2  27 (7%)  DCLRE1C  19 (5%)  IL7R  23 (6%)  CD3D/E/Z  3 (0.8%)  PTPRC  1 (0.3%)  PNP  1 (0.3%)  UNK/NR  152 (38%) | Median 5.8 mo (0.2 - 228 mo) | N=399  None  218 (55%)  MAC  82 (21%)  RIC  36 (9%)  IS  60 (15%) | N=399 Chronic GVHD  79 (19.8%) | Median 8.6 y (2 - 31.6 y) | N=399  **Neurologic**  34 (8.5%):  Motor disorder  11 (2.8%)  Hearing/speech/vision deficits  9 (2.3%)  Seizure  8 (2%)  Headache  3 (0.8%)  Cerebral palsy  2 (0.5%)  Infarction/TIA  2 (0.5%)  Neuropathy  1 (0.3%) Neurodevelopmental  31 (7.8%):  Global deficits  21 (5.3%)  Developmental delay  8 (2%)  Autism  2 (0.6%)  Dental  30 (7.5%)  **Pulmonary**  26 (6.5%):  Idiopathic pulmonary syndrome  15 (3.8%)  Clinically significant O2 requirement  9 (2.3%)  Bronchiolitis obliterans  4 (1.0 %)  Pulmonary hemorrhage  1 (0.3%)  **Musculoskeletal**  24 (6%)  **Hepatic**  18 (4.5%):  Clinically significant liver disease  16 (4%)  Severe transaminitis  14 (3.5%)  Autoimmune  15 (3.8%):  Vitiligo  6 (1.5%) Thrombocytopenia  3 (0.8%)  Neutropenia  3 (0.8%)  Myositis  2 (0.5%)  Alopecia  2 (0.5%)  Hemolytic anemia  1 (0.3%)  Arthritis  1 (0.3%)  Nephritis  1 (0.3%)  Endocrine  15 (3.8 %)  **Malignant**  **9 (2.3%):**  Lymphoma or lymphoproliferative  3 (0.9%)  Nonmelanoma skin malignancy  2 (0.6%)  Other malignancy  2 (0.6%)  Acute myeloid leukemia  1 (0.3%)  Leukemia (NOS)  1 (0.3%)  Myelodysplasia or myeloproliferative disorders  1 (0.3%)  Gastrointestinal  5 (1.3%)  Cardiac  2 (0.5%) |  |
| Golwala et al., 2023 (9) | Single-center, retrospective cohort study, United Kingdom  2000-2018 | Transplanted 429  Alive  340  Included in this review  340 | N=340  SCID  153 (45%)  CGD  42 (12.3%)  WAS  34 (10%)  CID  27 (7%)  Immunodeficiency with intestinal disorders  19 (5.5%)  XLP (excluding HLH as presentation)  12 (3.5%)  HLH (including XIAP and XLP)  16 (4.7%)  LAD/other neutrophil defect  11 (3.2%)  XIAP (excluding HLH as presentation)  2 (0.5%)  CD40 ligand deficiency  12 (3.5%)  DOCK8 deficiency  8 (2.3%)  IPEX  4 (1.1%)  APDS  3 (0.8%) | Not stated | N=340  Mean 33 mo (5 - 166 mo) | N=340  Treosulfan or Busulfan based conditioning  295 (87%)  Melphalan based conditioning  45 (13%) | N=32*  Acute GVHD  Grade  I/II 16(50%) grade  III/IV  5 (13%)  Chronic GvHD  9 (28%) | N=340  Mean 10.6 y (4.6 - 15 y) | N=340  non-osteopenic bone pathology  32 (9%)**  Out of them: Osteochondroma  10 (3%)  Genu valgum deformity  10 (3%)  Avascular necrosis  9 (3%)  Skeletal dysplasia  6 (2%)  Slipped upper femoral epiphysis  6 (2%)  Osteosarcoma  1 (0.3%)  Scoliosis  1 (0.3%)  Synostosis  1 (0.3%) | *GVHD described only in non-osteopenic bone pathology group  (N = 32)  **Some patients had more than one bone pathology: 11 (3%)  One patient (P27) was previously reported by Botto et al. (15). |
| Grunebaum et al., 2006 (29) | Two-center retrospective cohort, Canada, Italy  1990-2004 | Transplanted  105  Described in a study  94  Included in this review  56* | N=56  SCID  56 (100%) | N=56  γc (IL2RG)  12 (21%)  JAK3  10 (18%)  RAG1  6 (11%)  ADA  3 (5%)  RAG2  3 (5%)  IL7RA  2 (4%)  ARTEMIS  2 (4%)  FOXP3  1 (2%)  ZAP70  1 (2%)  RMRP  1 (2%)  CD38  1 (2%)  UNK/NR  14 (25%) | Infancy | N=94**  MAC  75 (80%) | N=56  Acute GVHD  34 (61%) Chronic GVHD  7 (9%) | N=56  > 2 years after HSCT | N=56  Neurologic deficits  3 (5%)  Recurrent respiratory infections  1 (2%)  Diplegia  1 (2%) | *Only data from patients who survived >2 y post HSCT were included in this study.  **Only data for the whole cohort is available. |
| Hardin et al., 2022 (17) | Single-center retrospective cohort, United States of America  1982-2019 | Transplanted: 177  Alive  129  Described in a study  88  Included in this review  88 | N=88  SCID  88 (100%) | N=88  X-linked  49 (56%)  ADA  9 (10%)  IL-7RR  10 (11%)  JAK3  7 (8%)  RAG1  3 (3%)  RAG2  3 (3%)  CD3  3 (3%)  ARTEMIS  1 (1%)  CD45  1 (1%)  CHH  1 (1%)  UNK/NR  1 (1%) | N=88  <3.5 mo  38 (43%)  >3.5 mo  50 (57%) | N=88  None  88 (100%) | Not explicitly stated | N=88  Median 13.9 y  (8 days - 38 y) | N=88  Immunoglobulin replacement  44 (50%)  Warts  36 (41%)  Sinus infections in the last 2 years  24 (27%)  Learning disability  21 (24%)  ADHD  21 (24%)  Anxiety  19 (22%)  On prophylactic antibiotics  18 (21%)  Developmental delay  11 (13%)  Depression  11 (13%)  Pneumonia in the last 2 years  8 (9%)  Underweight  6/82 (7%)* | *Only 82 patients reported their weight |
| Hönig et al., 2007 (18) | Two-centers  retrospective cohort/case series, Germany  Since 1982 | Transplanted  15  Alive >2 years  12  Included in this review 12 | N=12  SCID  12 (100%) | N=12  ADA  11 (92%)  Not confirmed  1 (8%) | N=12  Median 3.5 mo (2-17 mo) | N=12  None  7 (58%)  Bu-Cy  5 (42%) | N=12 Acute GVHD  2 (17%) Chronic GVHD  1 (8%) | N=12  Mean 12 y (4-22 y) | N=12  Motor dysfunction  7 (58%)  Learning disability  7 (58%)  Abnormal verbal expression  6 (50%)  Sensorineural hearing deficit  5 (42%)  Hyperactivity  4 (33%)  Seizures  1 (8%) |  |
| Lin et al., 2009 (25) | Single-center prospective longitudinal observational study, United States of America  1997-2006 | Transplanted  16  Described in a study  16  Included in this review  16 | N=16  SCID  16 (100%) | Not stated | N=16  Median 6.5 mo (2.5-14 mo) | N=16  Bu-based MAC  16 (100%) | Not stated | 1 y follow up  16 (100%)  3 y follow up  11 (69%) 5 y follow up  4 (25%) | Significant decline in cognitive and adaptive function at 1-year post-HSCT with stabilization by 3 years.  Psychomotor function declined later by 3 years (PDI 86.0 → 74.1, p = 0.008).  Younger age at transplant (<8 months) associated with greater adaptive decline (VABS 103.0 → 77.2 vs 97.0 → 85.8; p = 0.023) |  |
| Lum et al., 2021 (3) | Multicenter retrospective cohort study, Netherlands, United Kingdom  2009-2018 | Transplanted  596  Described in a study  596  Included in this review  596 | N=596  **SCID**  158 (27%)  **PID (non-SCID)**  438(73%):  CGD  82 (14%)  HLH  61 (10%)  MHC class II deficiency  33 (6%)  WAS  31 (5%)  DOCK8 deficiency  18 (3%)  CD40L deficiency  18 (3%) Other  353 (59%) | Genotype data were not provided for all patients, refer to the original article for full details. | N=596  Median 28 mo (0.5-220 mo) | N=596  MAC  123 (21%)  RIC  409 (68%)  None  64 (11%) | N=596 Grade I-II aGVHD 227 (38%)  Grade III-IV aGvHD 45 (8%)  Chronic GvHD 32 (5%) | N=596  Median 4.3 y (0.08 – 14.7 y) | N=596  **Non-hematological autoimmune disease 31 (5%):**  Autoimmune hypothyroidism  10/31 (32%)  Autoimmune thyroiditis  7/31 (22%)  Polyarthritis  3/31 (10%)  Graves’ disease 2/31 (7%)  Myositis  2/31 (7%)  SLE  1/31 (3%)  Guillain-Barre syndrome  2/31 (7%)  Nephritic/nephrotic syndrome 1/31 (3%)  Stiff person syndrome  1/31 (3%)  Transverse myelitis  1/31 (3%)  Optic neuritis 1/31 (3%) |  |
| Mazzolari et al., 2005 (26) | Retrospective single-center cohort, Italy  1991-2002 | Transplanted  11  Described in a study  11  Alive >2 years  9  Included in this review  9 | N=11  Omenn syndrome  11 (100%) | N=11  RAG1  7 (64%)  RAG2  1 (9%)  None  3 (27%) | N=11  Median 8.4 mo (4-18 mo) | N=11  Bu/CY/ATG/TT  1 (9%)  Bu/Cy/VP  1 (9%)  Bu/Cy/ATG  2 (18%)  BU/Cy/ATG/TT  1 (9%)  Bu/Cy/TT  2 (18%)  Bu/Cy  2 (18%)  Flu/Mel  1 (9%)  TT/ATG/Cy  1 (9%) | N=11  Acute GVHD  7 (64%)  No cases of chronic GVHD | N=9  Median 7.8 y (2.5-12 y) | N=9  Autoimmune hypothyroidism  1 (11%)  Myasthenia gravis  1 (11%)  Familial diplegia  2 (22%)*  Reduced vision 1 (11%)** | *Reported by authors as a complication unrelated to HSCT  **due to bilateral optic neuritis attributed to ethambutol used for disseminated BCG infection treatment. |
| Mazzolari et al., 2007 (8) | Single-center, retrospective observational cohort, Italy  1991-2002 | Transplanted  58  Alive  42  Described in the study  40  Included in this review  40 | N=58  Severe T-cell immunodeficiency  58 (100%) | N=40  JAK3  10 (25%) IL2RG  8 (20%) RAG1/2  6 (15%)  IL7R  3 (7.5%)  ARTEMIS  2 (5%)  FOXN1  1 (2.5%)  ADA  1 (2.5%)  RMRP 1 (2.5%)  Undefined 8 (20%) | N=40  Median 7 mo (1-34 mo)  Intrauterine transplantation:  3 | N=40  None  8 (20%)  ATG  2 (5%)  Bu/Cy  11 (28%)  Bu/Cy/ATG  4 (10%)  Bu/Cy/TT  4 (10%)  ATG/Cy/TT  1 (3%)  Bu/Cy/TT/ATG  3 (8%)  Flu/Mel/ATG  1 (3%)  Bu/Cy/VP16  1 (3%) | N=40  Grade 3 acute GvHD 4 (10%) | N=40  11 y (6-16 y) | N=40 Low weight ≤3rd percentile  7 (17.5%)  short stature ≤3rd percentile 5 (12.5%)  Endocrine abnormalities  7 (17.5%)  Severe neurologic problems  4 (10%)  Hearing abnormalities  2 (5%)  Ongoing immunoglobulin replacement  5 (12.5%)  Dental abnormalities  3 (7,5%) | Significant infections at >1 y after HSCT 5/40 (12.5)  Hospitalizations after first year after HSCT 8/40 (20%) |
| Mazzolari et al., 2009 (33) | Single-center, retrospective observational cohort, Italy  1991-2003 | Transplanted  74  Alive  53  Described in a study  49  Included in this review  49 | N=49  SCID  49 (100%) | N=49  JAK3  11 (22%)  IL2RG  10 (20%)  RAG  8 (16%)  IL7R  3 (6%)  ARTEMIS  2 (4%)  ADA  2 (4%)  RMRP  2 (4%)  FOXN1  1 (2%)  Reticular dysgenesis  1 (2%)  Genetically undefined  9 (18%) | Not stated | N=49  None  11 (22%)  MAC  36 (73%)  IS  2 (4%) | Not explicitly stated | N=49  125,1 mo (64,5-206,8 mo) | N=49 Papillomavirus warts  9 (18%)  Neurocognitive or sensorial problems  9 (18%)  Weight <3rd centile  7/49 (14%)  Length <3rd centile  4/49 (8%)  Autoimmune thyroid disease  5 (10%)  Autoimmune hemolytic anemia 4 (8%)  Other severe infections  4 (8%)  Interstitial pneumonia  3 (6%)  Glomerulonephritis  1 (2%) |  |
| Miyamoto et al., 2021 (11) | Retrospective cohort study, Japan  1974-2016 | Transplanted  181  Alive > 1 y after HSCT  127  Alive > 5 y after HSCT  110  Alive > 10 y after HSCT  107  Alive > 25 y after HSCT  80  Described in a study  181  Included in this review  181 | N=181  SCID  181 (100%) | N=181  IL2RG deficiency/X-linked  55 (30%)  JAK3 deficiency  3 (2%)  CD3 component deficiency  2 (1%) RAG1/RAG2 deficiency/Omenn  syndrome  14 (8%)  ADA deficiency  6 (3%)  Artemis deficiency  6 (3%)  DNA ligase IV deficiency 3 (2%)  Reticular dysgenesis  1 (0,5%)  Genetically undefined  91 (50%) | N=175  Median 7 mo (1-204 mo) | N=181  FLU/BU  28 (15%)  FLU/MEL  34 (19%)  None  72 (40%)  Flu/CY ATG  3 (2%)  Flu  2 (1%)  CY  1 (0.5%)  ATG  1 (0.5%)  Bu/CY  18 (10%)  Flu/CY/ETP/Mel 3 (2%)  Flu/Bu/Mel  2 (1%)  Flu low-dose TBI 2 (1%)  Flu/CY low-dose TBI  1 (0.5%)  TBI 8 Gy/CY  1 (0.5%)  AraC/ETP/CY  1 (0.5%)  Not explicitly stated  12 (7%) | Acute GVHD Grade III-IV 6/173 (3%)  Chronic GVHD 12/125 (10%) | N=181  3.7 y (1 day-28.5 y) | Short stature 24/110 (22%)  Hypothyroidism 4/56 (7%)  Gonadal dysfunction  3/33 (9%)  Non-hodgkin lymphoma  2/178 (1%)  Bronchiolitis obliterans  1/178 (1%) |  |
| Nagasawa et al., 2017 (31) | Retrospective single-center cohort study,  Japan,  2001–2011 | Transplanted  74  Alive  67  Included in this review  29* | N=29  WAS  9 (31%)  SCID  6 (21%)  Hyper-IgM syndrome  3 (10%)  CID  5 (17%)  Other  6 (21%) | Not stated | N=67  Median 6.5 y  (0.6 – 22.7 y) | N=67  MAC  49 (73%)  RIC  18 (27%) | N=67  Acute GVHD >II  34 (51%)  Acute GVHD >III  19 (28%)  Chronic GVHD  18 (27%) | N=67  Median 5.4 y (0.3 – 14 y) | N=67  LONIPCs  9 (13%)  Bronchiolitis obliterans  8 (12%)  BOOP  1 (2%) | *The article states that 29 patients had primary immunodeficiency; PID-specific late-effect data were not fully separable from the whole pediatric cohort. |
| Nahum et al., 2009 (27) | Prospective single-center cohort, Canada  1989-2007 | Transplanted  6  Alive  6  Described in a study 6  Included in this review  6 | N=6  Omenn syndrome  6 (100%) | N=6  RMRP 2 (33%)  T+B+NK+,  ligase 4  1 (17%)  Genetically undefined  3 (50%) | N=6  Median 9 mo (6-29 mo) | N=6  Bu/Cy 6 (100%) | N=6 Acute GVHD 6 (100%) Chronic GVHD 3 (50%) | N=6  Mean 7.9 y  (2-16 y) | N=6  Short stature 4 (67%)* | *Short stature is associated with cartilage hair hypoplasia (N=2) and ligase 4 deficiency (N=1).  One patient is under investigation for the cause of linear growth retardation. |
| Neven et al., 2009 (32) | Retrospective, single-center cohort study, France  1972-2004 | Transplanted  149  Alive > 2 y after HSCT  94  Described in a study  90  Included in the review  90 | N=90  SCID 90 (100%) | N=90  IL2RG (γc)  22 (25%)  RAG1/2  20 (22%)  JAK3  16 (18%)  Artemis  12 (13%)  IL7Rα  6 (7%)  Reticular dysgenesis  4 (5%)  ADA-SCID  3 (4%)  CD3ε  1 (1%)  UNK/NR 6 (7%) | N=90  ≤3.5 mo  23 (25%)  >3.5 mo  67 (75%) | N=90  None  46 (51%) Immunosuppression  5 (6%)  BU8/Cy 200 mg/kg  22 (25%)  BU16/Cy 200 mg/kg  17 (19%) | N=90  Acute GVHD ⪖grade 2  31 (34%)  Chronic GVHD  24 (27%) | N=90  Median  14 y (2-34 y) | N=90  Chronic HPV infection  23 (26 %)  Persistent digestive symptoms and requirement for nutritional support  18 (20%)  Weight or height <3rd percentile 16 (18%)  Chronic sinusitis/chronic bronchopneumopathy  6 (7%)  Autoimmune hemolytic anaemia  6 (7%)  Psychosocial disabilities  6 (7%)  Ill-defined inflammatory disease  3 (3%)  Opportunistic infection  3 (3%)  Obesity  3 (3%)  Myositis  2 (2%)  Myelodysplasia*2 (2%)  Recurrent LRTI 2 (2%)  Psoriasis, vitiligo,alopecia 1 (1%)  Disseminated granulomatous disease 1 (1%)  Viral encephalitis 1 (1%) | *Patients with reticular dysgenesis. |
| O’Marcaigh et al., 2001 (5) | Single-center retrospective case series, United States of America  1984-1999 | Transplanted  16  Alive > 2 y post HSCT  11  Included in the review  11 | SCID  11 (100%) | Not stated | Median 2.5 mo (1-5.5 mo) | N=11  Cy200/ATG80  8 (73%)  ATG  2 (18%)  Cy/ATG/BU  1 (9%) | No patient developed acute GVHD > grade 2 | N=12  At least >4 y (median 7.2 y) | N=11  Growth impairment  3 (28%)  Failure of secondary tooth development 2 (18%)  Restrictive lung disease  1 (9%)  Alveolar hemorrhage  1 (9%)  Late death  2 (18%)* | Late death associated with severe HUS (5 y 6 mo after HSCT) and severe AIHA and fungal pneumonia (7 y 5 mo) |
| Patel et al., 2008 (16) | Single-center, retrospective observational study, United States of America  1981-1995 | Transplanted  25  Alive  15  Described in a study 25  Included in the review  15 | N=25  SCID  25 (100%) | N=25  IL2RG  7 (28%)  RAG1  5 (20%)  UNK/NR  13 (52%) | N=15  Median 5 mo  (0.5-8 mo) | N=15  None 15 (100%) | N=15 Acute GVHD  15 (100%) Chronic GVHD 5 (20%) | N=15  Median 16.3 y (10-27 y) | N=15  Viral warts  5 (27%)  Gastrointestinal and hepatic disease  3 (20%)  Hearing impairment  3 (20%)  Bronchiolitis obliterans  2 (13%)  Asthma  2 (13%)  Short stature  2 (13%)  Neurological and neurodevelopmental complications  2 (13%)  Vitiligo  1 (7%)  Alopecia  1 (7%)  Autoimmune fasciitis  1 (7%)  Spongiotic dermatitis  1 (7%)  Bronchiectasis  1 (7%)  Pituitary microadenoma  1 (7%)  Adrenal Insufficiency  1 (7%)  Galactorrhea, amenorrhea  1 (7%)  Dental caries  1 (7%)  Myopia  1 (7%)  Blindness  1 (7%) |  |
| Patel et al., 2009 (22) | Single-center retrospective cohort, United States of America  1998-2007 | Transplanted  23  Alive  18  Included in the review  13* | SCID  13 (100%) | N=13  IL2RG  7 (54%)  RAG1  2 (15%)  IL7Rα  2 (15%)  ADA  1 (8%)  UNK/NR  1 (8%) | N=13  Median 4 mo  (0.75-23 mo) | N=13  None  3 (23%)  Bu/Cy/araC/ATG or C-1H, CSA/pred  6 (46%)  Flu/C-1H, ±CSA or FK  1 (8%)  Flu/TBI/C-1H, FK  1 (8%)  C-1H  1 (8%) | N=13 Acute GvHD  1 (8%) | N=13  Median 5 y (2.4-9.8 y) | N=13  Asthma  4 (31%)  Obesity  2 (15%)  IgA def  2 (15%)  Caries  1 (8%)  Warts  1 (8%)  HHV6 infection  1 (8%)  Cataracts  1 (8%)  Migraines  1 (8%)  Protein loosing enteropathy  1 (8%)  Hypersensitivity  1 (8%)  Reactive airway disease  1 (8%)  Allergic rhinitis  1 (8%)  Eczema  1 (8%)  Poor growth  1 (8%)  Speech delay  1 (8%) | The data reported in the text do not fully correspond with the values presented in the table. The figures used here were extracted directly from Table I.  *Only data from patients who survived >2 y post HSCT were included in this study. |
| Railey et al., 2009 (13) | Single-center retrospective cohort study, United States of America  1982-2008 | Transplanted  161  Alive  124  Described in the study  111  Included in this review  111 | N=161  SCID  161 (100%) | N=111  IL2RG  53 (48%)  ADA deficiency  16 (14%)  IL7Rα deficiency  15 (14%)  autosomal recessive unknown defect  8 (7%)  JAK3 deficiency  6 (5%)  RAG1/ RAG2 deficiency  6 (5%)  CD3 chain deficiency  4 (4%)  CD-45 deficiency  1 (1%)  Cartilage hair hypoplasia with SCID  1 (1%)  unknown molecular cause  1 (1%) | N=161  <3.5 mo  48 (30%)  >3.5 mo  113 (70%) | N=161  None  161 (100%) | N=111 Chronic GVHD  2 (2%) | N=161  Median 104 mo (6-312 mo) | N=111  Ongoing immunoglobulin replacement  59 (53%)  Persistent rashes  28 (25%)  ADHD  23 (21%)  Sinusitis  24 (22%)  Asthma  13 (12%)  Chronic diarrhea  11 (10%)  Weight <3rd percentile  12 (11%)  Height <3rd percentile  13 (12%)  Development delay  15 (14%)  Pneumonia  8 (7%)  Hypothyroidism  5 (5%)  Reccuring HPV infection  13 (12%)  GERD  7 (6%)  Otitis media  6 (5%)  Oral aversion  6 (5%)  Seizure disorder  2 (2%)  Cerebral palsy  2 (2%)  Intermittent hemolytic anemia  2 (2%)  Intermittent thrombocytopenia  1 (1%) |  |
| Roifman et al., 2008 (28) | Prospective single-center observational cohort, Canada  1989-2005 | Transplanted  10  Alive  10  Described in the study  10  Included in this review  10 | T⁺CID  10 (100%) | N=10  IL2RG (γc)  deficiency  4 (40%)  Omenn syndrome  (Gene not  specified)  4 (40%)  RMRP-associated Omenn  syndrome  1 (10%)  Cartilage hair hypoplasia  1 (10%) | Median 11.75 mo (6-59 mo) | N=10  Bu/Cy  10 (100%) | N=10  Any acute GVHD  10 (100%)  Chronic  GVHD  3 (30%) | Median  137.5 mo (28–192  mo) | N=10  Short stature  3 (30%)  (1 associated with cartilage hair hypoplasia) |  |
| Schwaderer et al., 2005 (20) | Case report, USA,  not stated | Transplanted  2  Alive  2  Included in this review  2 | SCID  2 (100%) | N=2  X-linked SCID  2 (100%) | First year of life | N=2  None  2 (100%) | Not confirmed  (possible low-grade delayed cGVHD considered) | Several years post-HSCT | N=2  Granulomatous interstitial nephritis  2 (100%)  Systemic granulomatous disease  2 (100%)  Granulomatous / plaque-like skin lesions  2 (100%)  Chronic renal insufficiency  1 (50%)  Pancytopenia  1 (50%)  Incomplete B-cell engraftment requiring IVIG  2 (100%) |  |
| Scott et al., 2017 (34) | Retrospective, single-center observational cohort, Canada  1985-2015 | Transplanted  14  Alive >5y  5  Described in the study  5  Included in this review  5 | SCID  5 (100%) | N=5  ADA-deficiency  5 (100%) | Not explicitly stated | N=5  None  3 (60%)  Bu/Cy  2 (40%) | Not stated | Survived >5 y | N=5  Gastric reflux  4 (80%)  Hearing impairment  3 (60%)  Global developmental delay  2 (40%)  Seizure disorder  2 (40%)  Chronic lung disease and bronchiectasis  2 (40%)  Tube feeding  2 (40%)  Skin eczema  2 (40%)  Cerebral palsy due to intracranial bleeding  1 (20%)  Hypertension  1 (20%)  Allergic rhinitis, sino-nasal polyps  1 (20%)  Neutropenia  1 (20%) Dermatofibrosarcoma protuberans  1 (20%)  Diffuse punctate keratitis  1 (20%)  Amblyopia  1 (20%)  Hypothyroidism  1 (20%)  Osteopenia and recurrent fractures  1 (20%)  Polycystic  ovarian syndrome  1 (20%)  Height/weight <3/3 percentile  1 (20%)  Weight < 3  percentile  1 (20%)  Recurrent infections  2 (40%) | Severe demyelinating disease after lung transplant  1 (20%) |
| Slatter, 2004 (4) | Single center, retrospective cohort, United Kingdom  1987-2002 | Transplanted  83  Alive  83  Described in the study  9  Included in this review  9 | N=83  SCID  49 (59%)  CID  9 (11%)  WAS  7 (8%)  CGD  11 (13%)  XL-HIM  5 (6%)  Osteopetrosis  2 (2%) | N=9  RAG2 deficiency  1 (11%)  IL2RG  1 (11%)  Artemis  1 (11%)  ADA deficiency  1 (11%)  JAK3 deficiency  2 (22%)  WAS  2 (22%)  Osteopetrosis  1 (11%) | N=9  Median 6 mo (1–72 mo) | N=9  None  1 (11%)  Bu/Cy/ATG  2 (22%)  Bu/CY  5 (56%)  Flu/Bu/Cy  1 (11%) | N=9  Acute  GVHD  4 (44%)  Chronic  GVHD  2 (22%) | Not explicitly stated | N=9  Compensated  hypothyroidism  7 (78%)  Primary hypothyroidism  2 (22%) |  |

Abbreviations: ADA – adenosine deaminase; ADHD – attention-deficit/hyperactivity disorder; AIHA – autoimmune hemolytic anemia; ALL - AML – acute myeloid leukemia; APDS – activated PI3Kδ syndrome; AVN – avascular necrosis; BMT – bone marrow transplantation; BU – busulfan-based conditioning; CGD – chronic granulomatous disease; CID – combined immunodeficiency; cGVHD – chronic graft-versus-host disease; DOCK8 – dedicator of cytokinesis 8; FOXN1 – forkhead box N1; GH – growth hormone; GVHD – graft-versus-host disease; HLH – hemophagocytic lymphohistiocytosis; HSCT – hematopoietic stem cell transplantation; IEI – inborn error of immunity; IEM – inborn error of metabolism; IL7Rα – interleukin-7 receptor alpha; JAK3 – Janus kinase 3; LAD – leukocyte adhesion deficiency; LTMAC – low-toxicity myeloablative conditioning; MAC – myeloablative conditioning; NOS – not otherwise specified; PID – primary immunodeficiency; RAG – recombination activating gene; RIC – reduced-intensity conditioning; RMRP – RNA component of mitochondrial RNA processing endoribonuclease; SCID – severe combined immunodeficiency; UNC – unconditioned; UNK/NR – unknown or not reported**;** VP – etoposide; WAS – Wiskott–Aldrich syndrome; XIAP – X-linked inhibitor of apoptosis; XLP – X-linked lymphoproliferative disease.
