# Supplementary material for: Late effects after allogeneic hematopoietic stem cell transplantation in patients with primary immunodeficiency
Source: Front Immunol. 2026 Jul 1;17:1752700. doi: 10.3389/fimmu.2026.1752700 (PMC13368574; doi:10.3389/fimmu.2026.1752700)
Supplement: Supplementary Figure 1 — Late effects across the largest and most comprehensively reported pediatric PID HSCT cohorts. [file DataSheet1.docx]

**Supplementary Figure 1.** Late effects across the largest and most comprehensively reported pediatric PID HSCT cohorts. This figure depicts only selected studies to enable clear visualization of late-effect patterns in cohorts with comparatively large numbers of survivors with ≥2-year follow-up and multi-domain late-effect reporting. Including: Eissa et al. 2024 (N=399), Railey et al. 2009 (N=111), Neven et al. 2009 (N=90), Hardin et al. 2022 (N=88), Miyamoto et al. 2021 (N=181).

A

B
